# Interconversion of Cancer Cells and Induced Pluripotent Stem Cells

**DOI:** 10.3390/cells13020125

**Published:** 2024-01-10

**Authors:** Drishty B. Sarker, Yu Xue, Faiza Mahmud, Jonathan A. Jocelyn, Qing-Xiang Amy Sang

**Affiliations:** 1 Department of Chemistry and Biochemistry, Florida State University, Tallahassee, FL 32306-4390, USA; ds22@fsu.edu (D.B.S.); yx21@fsu.edu (Y.X.); fm23@fsu.edu (F.M.); jaj21g@fsu.edu (J.A.J.); 2 Institute of Molecular Biophysics, Florida State University, Tallahassee, FL 32306-4380, USA

**Keywords:** induced pluripotent stem cells, cancer stem cells, reprogramming, pluripotency, interconversion, cancer stem cell models, induced pluripotent cancer cells

## Abstract

Cancer cells, especially cancer stem cells (CSCs), share many molecular features with induced pluripotent stem cells (iPSCs) that enable the derivation of induced pluripotent cancer cells by reprogramming malignant cells. Conversely, normal iPSCs can be converted into cancer stem-like cells with the help of tumor microenvironment components and genetic manipulation. These CSC models can be utilized in oncogenic initiation and progression studies, understanding drug resistance, and developing novel therapeutic strategies. This review summarizes the role of pluripotency factors in the stemness, tumorigenicity, and therapeutic resistance of cancer cells. Different methods to obtain iPSC-derived CSC models are described with an emphasis on exposure-based approaches. Culture in cancer cell-conditioned media or cocultures with cancer cells can convert normal iPSCs into cancer stem-like cells, aiding the examination of processes of oncogenesis. We further explored the potential of reprogramming cancer cells into cancer-iPSCs for mechanistic studies and cancer dependencies. The contributions of genetic, epigenetic, and tumor microenvironment factors can be evaluated using these models. Overall, integrating iPSC technology into cancer stem cell research holds significant promise for advancing our knowledge of cancer biology and accelerating the development of innovative and tailored therapeutic interventions.

## 1. Introduction

Induced pluripotent stem cell (iPSC) technology has been the cornerstone of stem cell research and regenerative medicine since its emergence [1]. The long quest for a more accessible and robust pluripotent cell resource culminated in the breakthrough of pluripotency induction in somatic cells by Yamanaka and Takahashi in 2006 using a combination of four transcription factors—Oct4, Sox2, c-Myc, and Klf4—that are also referred to as “Yamanaka factors” [2]. The demonstration thereby supplanted the need for controversial embryonic stem cell (ESC) sources for generating differentiated human cells of diverse lineages [3,4]. Over the past decade and a half, iPSCs have been extensively utilized in scientific studies due to their enormous scope in cell therapy [5,6,7,8] and disease modeling [9,10] for mechanism research and drug discovery.

Cancer is a generic name for a constellation of malignancies characterized by uncontrolled growth and division [11]. At the origin of cancer is an abnormal cell that acquires either a causative mutation or causative mutations before undergoing oncogenic transformation [12]. The global cancer burden is on the rise, and addressing the high-mortality epidemic requires expedited translational research powered through an expanded availability of suitable cancer models. The promise of iPSC technology in cancer modeling has been substantiated through abundant methods for introducing genetic and epigenetic changes in cells that can drive malignant transformation [13,14,15]. Such models are advantageous in studying cancer initiation and altered pathways related to progression and metastasis [16,17,18]. Moreover, cancer dependencies can be discovered with the help of iPSC-derived cancer avatars, thereby aiding the identification and validation of therapeutic targets for anticancer drug development [19].

In addition to performing oncogenic manipulation in normal iPSCs, pluripotency induction in malignant or pre-malignant cells can generate cancer stem cell (CSC) models [20,21]. CSCs are considered tumor-initiating cells with a high self-renewal and differentiation potential, like that of iPSCs, and are involved in cancer therapeutic resistance and relapse [22]. Therefore, the intertwined aspects of pluripotency and malignancy can be explored at large in iPSC-derived cancer models for a comprehensive understanding of CSC behavior and effective cancer therapies. On the contrary, some cancer-derived induced pluripotent stem cells exhibit reduced malignancy themselves and further differentiate into benign cells of distinct lineages [23,24]. The outcome of reprogramming thus seems to be dictated by an intricate relationship between genetic and epigenetic determinants in cancer cells—a deconvolution of which may provide valuable insight into oncogenic processes and strategies to modulate them.

This review first discusses the highly similar molecular characteristics of iPSCs and cancer cells with an emphasis on pluripotency genes. The origin and biology of cancer stem cells are then reviewed as the malignant counterpart of normal pluripotent stem cells. Finally, a detailed account of the interconversion of cancer cells and iPSCs is presented to highlight the challenges and opportunities regarding the application of iPSC technology in cancer research.

## 2. Shared Molecular Features between iPSCs and Cancer Cells

**Figure 1:**
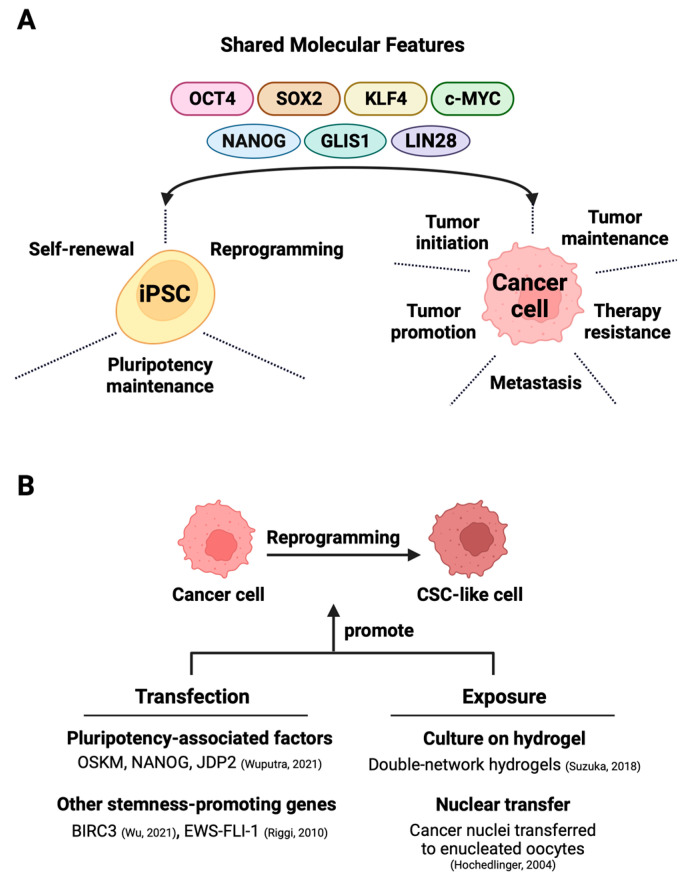
Exploiting shared molecular characteristics between iPSCs and cancer cells for the generation of cancer stem cells. (**A**) The shared factors not only support the self-renewal, pluripotency, and maintenance of iPSCs but also partake in oncogenesis in various cancers (details in Section 2.1). (**B**) The direct reprogramming of bulk cancer cells into cancer stem cells can occur through two main methods—one involves techniques like that of transfection that induces the expression of stemness-promoting proteins [25,26] (details in Section 3) or triggers factor-dependent reprogramming [27] (details in Section 5). The alternative exposure-based method relies on hydrogel-activated reprogramming [28] or nuclear transfer techniques [29] to drive CSC formation (details in Section 5).

The application of iPSC technology in cancer research benefits from a high extent of shared molecular features between iPSCs and cancer cells (Figure 1A). This section explores the parallel molecular signatures and signaling pathways commonly manifested between iPSCs and cancer cells.

### 2.1. The Role of the Four De Facto Pluripotency Inducers in Cancer Cells

Octamer-binding transcription factor 4 (Oct4), also known as Oct3 or POU5F1 (POU domain, class 5, transcription factor 1), is a prominent regulator of the induction and maintenance of cellular pluripotency and is capable of reprogramming neural stem cells to pluripotency individually [30]. Oct4 overexpression, although circumstantial, has been linked to tumorigenesis in gastric cancer cells [31] and lung adenocarcinoma [32]. Additionally, Oct4 has been observed to play a role in the maintenance and progression of tumors in breast cancer [33], nasopharyngeal carcinoma [34], bladder cancer [35], rectal cancer [36], brain cancer [37], and ovarian cancer [38] as well as in chemoresistance in bladder cancer [39]. Oct4 predominantly exerts its functions through the formation of complexes with other proteins, such as Sox2, Nanog, beta-catenin, and others. Given its participation in multiple pathways associated with tumorigenesis and tumor maintenance, Oct4 may emerge as a promising target for cancer treatment strategies [40].

SRY (sex-determining region Y)-box 2, also known as Sox2, is another crucial factor for pluripotency induction in human somatic cells, and its cooperativity with Oct4 is instrumental for its function [41]. Sox2 overexpression has been linked to oncogenic initiation, amplification, and maintenance in ovarian [38], lung [42], and pancreatic [43] cancers. It has been observed to play a role in late carcinogenesis in prostate [44] and pancreatic cancer [45] as well as in chemoresistance in prostate cancer [44].

Transcription factor Krüppel-like factor 4 (Klf4) interacts directly with Oct4 and Sox2 to activate Nanog, which is another transcription factor that is responsible for the maintenance of pluripotent cells in the inner cell mass of blastocysts by blocking stem cell differentiation [46,47]. Unlike the other pluripotency genes, Klf4 has been observed to possess a tumor-suppressive role in gastric [48] and colorectal [49] cancers. However, its oncogenic role is indicated in the cases of breast cancer [50] and squamous cell carcinoma of the esophagus [51]. The diverse functions exhibited by Klf4 likely stem from its distinctive structure that encompasses both transcriptional activation and repression domains. Furthermore, numerous proteins associated with tumorigenesis, such as p21, p27, p53, and Cyclin D, are downstream targets of Klf4. Its involvement in inflammation and precancerous lesions adds to its significance in tumorigenesis studies [52].

Transcription factor c-Myc enhances pluripotency to generate high-quality iPSCs along with the previously mentioned factors [53]. Overexpression of c-Myc has been tied to prostatic neoplasia [54], and the inactivation of its antagonist tumor suppressors BRCA1 and SMARCB1 has been observed to result in its overexpression in breast cancer [55] and malignant brain rhabdoid tumors [56], respectively. Conversely, c-Myc inactivation has been shown to cause the regression of liver tumors, corroborating its role as an oncogene [57].

### 2.2. The Role of Auxiliary Pluripotency-Related Factors in Cancer Cells

Despite being related to Klf4, which has known tumor-suppressive properties, Nanog has been linked to the promotion of tumor growth in breast cancer [58] as well as chemoresistance and regenerative capacity in prostate cancer [59]. It has also been observed to be overexpressed in hypoxia-induced aggressiveness of prostatic and pancreatic cancers [60,61].

Glis family zinc finger 1 (Glis1) is a pro-reprogramming factor that acts in collaboration with the main pluripotency inducers [62,63]. Overexpression of Glis1 has been reported in breast cancer cells where it enhances cell migration and invasion capacity together with CUX1 [64]. A similar role of the protein is observed in ovarian cancer cells as well [65].

LIN28a/b (LIN28) is an RNA-binding protein that regulates development-associated genes post-transcriptionally [66]. It has been shown to enhance reprogramming efficiency [67] and has been linked to the aggressiveness of esophageal cancer [68], the initiation and maintenance of liver cancer [69], chemoresistance in breast cancer [70], and the suppression of the p53 tumor suppressor gene [71].

The shared features between iPSCs and cancer cells are summarized in Table 1.

## 3. Cancer Stem Cells

Cancer stem cells (CSCs) constitute a small subset of cells within tumors, demonstrating the capacity for self-renewal, differentiation, and the initiation of tumor formation upon transplantation into an animal host [22]. They also display a higher expression of drug efflux pumps and elevated DNA repair activity [72]. These properties position CSCs as the primary contributors to resistance against chemotherapy and radiotherapy as well as the recurrence and relapse of cancer [22,72].

### 3.1. Theories of Cancer Stem Cell Origin

The origin of the CSC concept can be traced back to 1875 when Julius Cohnheim et al. proposed the “embryonal rest” theory, which suggests that cancerous growth may arise from residual embryonic cells persisting after development and remaining dormant until activation [73,74,75]. The initial modern identification of CSCs occurred through a CD34+/CD38− subpopulation of malignant cells being isolated from human acute myeloid leukemia (AML) [76]. This specific cell type demonstrated the ability to generate colony-forming progenitors when engrafted into SCID mice. Over time, various CSC biomarkers have been identified for different cancers, including breast, prostate, brain, stomach, liver, and others [77].

CSC genesis within tumors remains a point of contention. The “progenitor origin model” postulates that CSCs emerge from adult stem or progenitor cells undergoing carcinogenesis due to accumulated mutations [78], with some retaining stemness characteristics while others differentiate. This model complies with the “hierarchy model”, which states that tumors comprise a large number of differentiated or differentiating bulk cancer cells without proliferative capacity and a small population of CSCs that give rise to the bulk cells through division and subsequent differentiation [79]. The progenitor hypothesis identifies CSCs as the cells of cancer’s origin, but some argue that CSCs are instead “cancer-propagating” cells rather than “cancer-initiating” cells in the original tumor [12]. Alternatively, the “stochastic model” of CSC origin suggests that CSCs arise de novo from any cancer cell in the tumor under appropriate microenvironmental cues [79]. The hierarchical and stochastic models are successful in explaining the characteristic tumor architectures of disparate cancers [76,80,81]; their unification is as yet elusive in the context of CSC origin.

### 3.2. Cellular Plasticity in Cancer Cells

Cancer cell plasticity is a fundamental concept of the stochastic model of CSC origin and enables the acquisition of stem cell features by bulk cancer cells. In 2013, Ischenko et al. showed that the conditional expression of oncogenic KrasG12D in non-stem mouse cells resulted in the emergence of stemness features and metastatic potential. Subsequent analysis revealed c-Myc as a determining factor in the transformation of KrasG12D-expressing cells [82]. Further supporting the idea, Schwitalla et al. demonstrated a positive correlation between NF-κB signaling and the Wnt pathway that drives the dedifferentiation of colon cancer cells [83]. More recently, BIRC3 overexpression has been linked to enhanced self-renewal and stemness maintenance in glioblastoma cell lines and patient-derived glioblastoma cells [25].

In addition, microenvironmental signals also contribute to the generation of CSCs. Vermeulen et al. reported the impact of myofibroblast-secreted factors, such as the hepatocyte growth factor (HGF), in amplifying Wnt signaling activity and consequently triggering the formation of CSCs in colon cancer cells [84]. In an unexpected revelation, Landsberg et al. discovered that proinflammatory cytokine tumor necrosis factor-alpha (TNF-α) could facilitate the dedifferentiation of melanoma cells, leading to resistance against cytotoxic T cells [85].

### 3.3. Pluripotency-Associated Genes in Cancer Stem-like Feature Acquisition

Elucidating the role of pluripotency-associated genes in CSC formation and malignant activity is critical for obtaining desirable cancer stem-like cells in vitro. In 2010, Riggi et al. discovered that the *EWS*-*FLI-1* fusion gene, the primary driver of Ewing sarcoma, could stimulate the expression of oncogenes SOX2, OCT4, and NANOG in human pediatric mesenchymal stem cells. Among these factors, SOX2 emerged as the pivotal element, with its lone expression capable of inducing CSC features in a primary tumor [26]. Yin et al. later demonstrated that the co-expression of OCT4 and NANOG in hepatocellular carcinoma (HCC) accelerated the epithelial–mesenchymal transition (EMT) process through the STAT3/Snail signaling pathway. The transfected HCC cells acquired CSC traits, including self-renewal, drug resistance, high tumorigenicity, and increased proliferation [86].

Recently, Liu et al. showed that biomechanical forces facilitate the interaction between TAZ and NANOG leading to the upregulation of SOX2 and OCT4, thereby enhancing CSC properties in human breast cancer [87]. Qi et al. previously identified a strong association between KLF4 and the stemness of human osteosarcoma cancer cells. Overexpression of KLF4 resulted in an increased sphere-forming potential, elevated expression of stemness genes, and heightened metastatic potential, with the p38 MAPK pathway implicated in the cell transition [88]. Kim et al. demonstrated that c-MYC could promote stemness and tumorigenicity in triple-negative breast cancer by inhibiting tumor suppressor zinc finger transcription factor 148 (ZNF148) [89]. Additionally, high c-MYC activity was observed in CD133+ colon CSCs, and the knockdown of c-MYC significantly attenuated CSC properties both in vitro and in vivo [90].

## 4. Deriving Cancer Stem-like Cells from iPSCs

Conversion of iPSCs into cancer stem-like cells can be achieved through the genetic manipulation of the iPSCs [14,91]. Given the lethal effect of some oncogenic mutations on iPSCs [91,92], inducible gene expression systems are widely used to obtain progenitor cells with CSC characteristics. Such systems are extremely useful for studying the stage- and tissue-specific oncogenicity of cancer-predisposing mutations in early carcinogenesis events. However, as previously discussed, the cell origin of cancer may not be identical to cancer-propagating or cancer-initiating CSCs [12]. Therefore, iPSCs with cancer-causing mutations are more useful for cancer initiation studies than CSC modeling. Yet, a handful of studies, mostly focusing on brain tumor modeling, reported cancer stem cell-like properties in engineered/edited iPSC-derived stem and progenitor cell types. Haag et al. showed that the H3.3-K27M mutation in iPSC-derived neural stem cells led to stemness maintenance and increased proliferation through the gliomagenic cells [91], while an earlier study by Koga et al. demonstrated the high self-renewal capacity of *PTEN*^−/−^; *NF1*^−/−^ and *TP53*^−/−^; *PDGFRA*^Δ8–9^ iPSC-derived high-grade glioma (HGG) spheres [14]. In 2012, Friedmann-Morvinski et al. conducted a study on mouse models where they introduced a lentiviral construct carrying two shRNA sequences against *NF1* and *TP53* genes into astrocytes and neurons to dedifferentiate the cells into glioblastoma multiforme (GBM)-forming neural progenitor/stem-like cells [93]. Though not relating to iPSCs, this study provides strong evidence for CSC induction through the conditional manipulation of gene expression even in differentiated cells.

**Figure 2:**
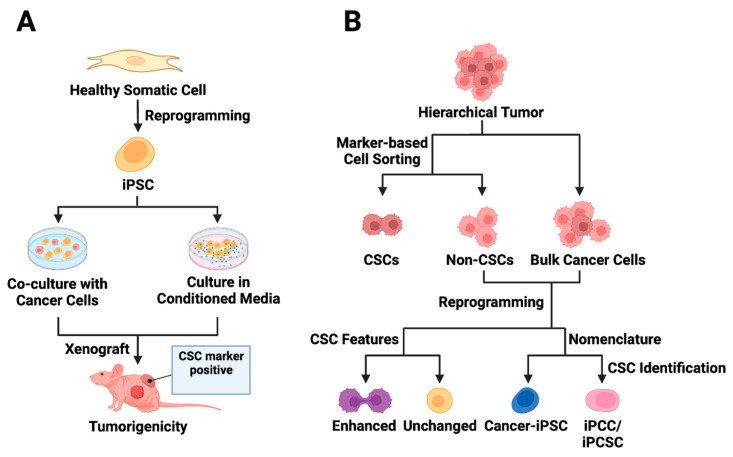
Utilizing iPSC technology in cancer stem cell modeling. (**A**) Exposure-based approaches to CSC model derivation from iPSCs are presented. The first approach involves co-culturing normal iPSCs with cancer cells, while the second strategy entails culturing iPSCs in conditioned media from cancer cells. The iPSCs exposed to tumor microenvironment factors acquire distinct CSC features and demonstrate the ability to form tumors in vivo. (**B**) The reprogramming of cancer cells is depicted, acknowledging the hierarchical structure of tumors. Cell sorting enables the separation of CSC marker-expressing cells from bulk tumor cells, though the presence of CSC markers may not always correlate with CSC features (non-CSCs). Following the reprogramming of non-stem cancer cells, some cancer-derived iPSCs (cancer-iPSCs) may exhibit enhanced CSC features. The reprogrammed cell population is called cancer-iPSCs, induced pluripotent cancer cells (iPCCs), or induced pluripotent cancer stem cells (iPCSCs).

Exposure of normal stem cells to malignant niches has been proposed as a model for CSC origin [94,95,96]. Conforming to this hypothesis, CSC models were generated in vitro either by exposing normal iPSCs to conditioned media (CM) from cancer cells of different tissue origin [13,97,98,99,100] or by coculturing the iPSCs with the cancer cells [13] (Figure 2A). A seminal study involving various mouse cancer cell lines demonstrated that CM-dependent CSC derivation from mouse iPSCs (miPSCs) was more successful than the coculture method [13]. The CM-exposed miPSCs generally exhibited CSC-like properties by manifesting self-renewal in sphere-formation assays and forming tumors when transplanted into nude mice [13,97,98,99,100]. A noteworthy observation is that tumors derived from different CM-based CSCs exhibit varying angiogenic and metastatic potential apparently due to the difference in the expression of metastasis and angiogenesis regulators, such as IFNγ and MMPs, in corresponding CSCs [13].

CM-based CSCs can be differentiated into various stromal cells that bolster tumor growth. Subpopulations of CM CSCs have been shown to express VEGF-A [97,99] and FGF2 [99] angiogenic factors in vitro and in vivo to mediate the differentiation and maturation of CSC-derived endothelial cells through paracrine signaling [99]. Furthermore, iPSC-derived CSC-like cells have also been reported to give rise to cancer-associated fibroblasts [100], supporting tumor maintenance and CSC survival [101]. Tumor-associated myoepithelial cells can also arise from these models upon mammary fat pad transplantation [102].

The long-term tumorigenicity of these CSC models remains largely uncharacterized. However, serial transplantation of CSCs induced using pancreatic carcinoma-conditioned media into mice was shown to form more aggressive cancers with the downregulation of the CSC markers of CD133, CD24a, and EpCAM [98]. miPSCs exposed to Lewis lung carcinoma (LLC)-CM were shown to harbor a hypomethylated genome with an enhanced PI3K–Akt pathway [103] that was retained by subsequent progenies. It is important to note that the PI3K–Akt pathway has been reported as highly necessary for the viability of human iPSCs [104]. Later analysis uncovered prostaglandin E2 (PGE2) enrichment in LLC-CM and a higher conversion of miPSCs into CSCs with PGE2 as an additive in the culture media [105]. In addition, extracellular vesicles from LLC cells have been shown to render miPSCs capable of forming tumors in vivo [106]. Exposure to the E-cadherin-Fc chimera protein was also shown to promote CSC features in colon cancer cells [107].

A higher degree of plasticity distinguishes cancer stem cells from bulk cancer cells [108], and the distinction is particularly relevant in the context of the CM-dependent generation of malignant cells from iPSCs. A recent study on the U87MG glioblastoma cell line revealed that CM from bulk cancer cells and cancer stem cell-generated neuron-like glioblastoma cells and spherical glioblastoma stem cells from iPSCs, respectively. While both the induced cell types showed the MGMT, GLI2, LEF1, and β-catenin overexpression characteristic of glioblastoma, only the cancer stem-like cells expressed stem cell markers CD133 and CD44. This finding emphasizes CM compositional variation as a critical determiner of the molecular features of the induced cells [109].

An expedited in vivo approach to converting miPSCs into CSCs entails mixing cancer cells with iPSCs before injecting them into immunocompromised mice. Pancreatic cancer stem cells generated through this method showed varied morphology (mesenchymal-like vs. epithelial-like), tumorigenicity, differentiation capacity, drug sensitivity, and gene expression depending on the distinct microenvironment provided by different cancer cell-CM. However, generated CSCs usually exhibit enhanced invasion ability as well as upregulated energy production and cancer-related pathways compared with the parental iPSC lines [110].

Finally, the differentiation of iPSCs into cervical reserve cells using small molecules has been described. These reserve cells possibly give rise to cervical cancer stem cells [111].

### Tumorigenicity and Tumor-Promoting Potential of Other iPSC-Derived Cells

In an in vitro pancreatic ductal adenocarcinoma (PDAC) model, iPSC-derived stellate cells were cocultured with patient-derived organoids or cancer cells to mimic tumor stroma. The stellate cells promoted the growth of some organoids and cells but not all. The observation suggests that the growth of cancer cells depends on the properties of the original tumor and is differentially influenced by a given stromal cell type [112].

A wealth of reports has described the generation, application, and overall usability of iPSC-derived mesenchymal stem cells (iMSCs) for tissue regeneration, cancer therapy, and the treatment of immune-related diseases [113,114,115,116,117]. However, the oncogenicity of iMSCs, especially iMSCs derived from mutation-carrying iPSCs, requires careful investigation. A study showed that iMSCs derived from *BRCA*+/− iPSCs showed elevated expressions of VEGF, PDGF, and ANGPT angiogenic factors and promoted the formation of an extended vascular network both in vitro and in vivo. The haploinsufficient mesenchymal stem cells were also characterized by higher migration ability and significantly upregulated Periostin expression compared with its *BRCA*+/+ counterpart. An enhanced tumorigenic and metastatic potential of *BRCA*+/− iMSCs was also observed when co-injected with 4T1 breast cancer cells into the mammary tissue of NOD-SCID mice [17]. Promisingly, iMSCs derived from disease-free iPSCs are less protumorigenic, while bone marrow-derived mesenchymal stem cells tend to support tumor growth and invasion by producing PGE2, IL6, and other protumor factors [118,119]. It will be worthwhile to examine the basis of the dissimilar cancer-promoting properties between iMSCs and mesenchymal stem cells from other sources [120,121,122].

Table 2 summarizes the derivation of cancer stem-like cells from iPSCs without the need for genetic manipulation.

## 5. Reprogramming Cancer Cells to Obtain Cancer Stem-like Cells

With the advent of iPSC technology, it has been possible to convert patient-derived somatic cells with cancer-predisposing germline mutation into iPSCs for disease modeling and therapy evaluation [123,124,125]. Beyond these goals, directed dedifferentiation of malignant cells can yield CSC-like cells (Figure 2B) for cancer research. The direct reprogramming of non-stem bulk cancer cells into CSC-like cells can occur through the transfection of either CSC-promoting protein-coding genes [26,82] or pluripotency-associated genes, which is discussed in great detail in this section. An alternative method for direct reprogramming utilizes exposure-based approaches to drive CSC formation by cancer cells (Figure 1B).

Relying on reprogramming factors to induce pluripotency, an attempt to reprogram colorectal cancer cell lines with the Yamanaka cocktail yielded cancer-iPSCs that could differentiate into three germ layer lineages. Nevertheless, the down-regulation of pluripotency genes and a distinct miRNA profile suggest incomplete or partial reprogramming toward pluripotency. Additionally, the cancer-iPSCs attained an epithelial/mesenchymal hybrid phenotype owing to dysregulated miRNA expression [126].

Cancer cell reprogramming has proved an effective tool in translational medical research on myeloproliferative disorders. iPSCs derived from malignant cells of juvenile myelomonocytic leukemia (JMML) through lentiviral OSKM expression showed higher proliferative capacity in cultures and produced myeloid cells of pathological features upon differentiation [20]. More JMML models have been established after with the help of iPSC technology to facilitate the study of disease mechanisms and potent therapeutics [127,128]. iPSCs generated from imatinib-sensitive chronic myelogenous leukemia (CML) patients showed imatinib insensitivity despite restored the expression of the BCR-ABL oncoprotein. Hematopoietic differentiation of the CML-iPSCs regained sensitivity to imatinib, though a fraction of immature cells were still resistant to kinase inhibitors like CML-iPSCs [129]. Similar results were obtained for KBM7 leukemia cell line-derived iPSCs [130]. Recently, a streamlined OSKM-based reprogramming method has been described to derive iPSCs from genetically diverse AML patients, which can model the disease reliably upon xenotransplantation [131]. AML-iPSCs have previously been shown to lose the epigenetic memory of parental cells and lack oncogenic potential. However, the hematopoietic differentiation of these cells reinstates leukemic properties [132]. Cancer-iPSCs that retain oncogenic mutations and malignant properties of source cells thus present a robust and renewable source of in vitro models for drug screening and mechanistic studies of hematological malignancies [20,133,134,135,136,137].

Toward the derivation of brain CSC models, pluripotency induction using OCT4 and JDP2 reprogramming factors conferred DAOY medulloblastoma cell lines with higher in vivo tumorigenicity [27]. The enhancement of tumor formation potency may partly be explained by mTOR activation through epigenetic rewiring due to increased Oct4 [138]. Moreover, a study on DAOY, D341, and D283 medulloblastoma cell lines characterized undifferentiated cell subpopulations in each cell line phenotypically and functionally to conclude that D283 has the highest level of CSC enrichment among the medulloblastoma cell lines [139]. Genetic alterations and the position of the cell of tumor origin within the differentiation hierarchy may account for the observed heterogeneity of stemness features in cancer cell lines [140]. Glioblastoma cell lines T731 and T653 were reprogrammed with Oct4, Sox2, and Klf4 to derive induced pluripotent cancer cells, and the use of a small molecule, PD98059, was shown to increase reprogramming efficiency [141]. Fully reprogrammed iPSCs have been generated from plexiform neurofibroma cells with mutations in the *NF1* tumor suppressor gene, potentiating faithful modeling of the developmental tumor [142,143].

Melanoma cell lines have been widely studied for CSC generation. R545 murine melanoma cells were reprogrammed with Oct4, Klf4, and c-Myc pluripotency inducers to obtain non-tumorigenic cancer-iPSCs. Chimeric mice embodying R545-iPSC-derived cells did not develop tumors at five months [24]. In a later work, constitutive expression of OCT4, KLF4, and SOX2 in BRAF-mutant HT-144 human melanoma cells produced metastable iPSCs with reduced tumorigenicity and therapeutic resistance against MAPK inhibitors. These cancer-iPSCs could differentiate into neurons and fibroblast-like cells beyond melanocyte lineages due to the programming-induced resetting of the melanoma epigenetic memory [144]. Apart from pluripotency factor-based reprogramming, the transfer of R545 cell nuclei into enucleated mice oocytes led to the development of pluripotent embryonic stem cells with the potential to generate nuclear transfer chimeras. However, the chimeric mice developed multiple myeloma lesions at a very early age [29].

Reprogramming various human gastrointestinal cancer cell lines with OSKM factors produced cancer-iPSCs with lower proliferation in vitro. The differentiated cancer-iPSCs showed a decreased tumorigenic potential in vivo. These iPSCs could also undergo differentiation into cell lineages from all three germ layers and showed higher sensitivity to anticancer therapies [23]. However, the introduction of OSK into SW480 colon cancer cell lines grown on a serum-containing medium produced CSCs with high dye-efflux activity, self-renewal capacity, and sustained tumorigenicity in serial transplantation [21]. Hep3B liver cancer cells reprogrammed with four factors expressed pluripotency markers and showed stemness that declined over time [145]. The reprogramming of the PLC/PRF/5 hepatoma cell line yielded tumorigenic cells with aggressive phenotypes [146].

A study on PANC-1 human pancreatic cancer cells revealed that the CSC subpopulation with higher c-MET expression is more amenable to OSKM-based reprogramming [147]. A previous reprogramming work on the same cell line reported a regression in tumorigenic potency of reprogrammed cancer cells upon four factor transduction [148]. Concerning patient-derived pancreatic tumors, an iPSC line derived from primary PDAC cells showed pluripotency features and progressed from early to late invasive stages of PDAC upon differentiation [149]. Chiou et al. demonstrated that co-expression of Oct4 and Nanog in A549 lung adenocarcinoma cells led to an augmentation of the CD133+ subpopulation, intensifying their capabilities in EMT, drug resistance, and tumor initiation [150].

In addition to transcription factor-mediated reprogramming, various novel methods have emerged for manipulating the differentiation stage of cancer cells. In 2021, Suzuka et al. discovered that double-network hydrogels (DN gel), comprising PAMPS and PDMAAm, could swiftly reprogram brain cancer cells into CSCs. The DN gel interacts with surface proteins, leading to differential spatiotemporal activation of protein kinases and, consequently, the induction of stemness protein expression, such as GLI1. The interface between the gel and cells hosts protein complexes produced by the cells, creating an optimal niche for the maintenance of CSCs [28]. Thus, this technology allows for the isolation of CSCs or the reversion of cancer cells into a stem cell state.

Table 3 lists the findings from the studies aimed at pluripotency induction in cancer cells.

## 6. Discussion

Induced pluripotent cancer stem cells (iPSCs) have added significantly to the understanding of cancer research by providing a robust source of in vitro cancer models for the study of cancer initiation, progression, metastasis, and therapeutic vulnerabilities. Their striking similarities with cancer cells at molecular levels, high proliferative capacity, and tumor formation potency have stimulated a more direct use of iPSCs in the given area of research. The stem cell model of cancer propagation emphasizes the presence and active role of cancer stem cells (CSCs) in malignant tumor progression and therapy resistance. The “stemness” properties shared between CSCs and iPSCs thus fueled the characterization of the tumorigenic potential of different iPSC lines. Moreover, the CSC-like behavior of iPSCs in response to tumor microenvironment (TME) components and cancer cell reprogramming to obtain CSC-like cells present areas of growing research interest.

So far, the most feasible way for converting iPSCs into cancer stem cells is the exposure of iPSCs to conditioned media (CM) from cancer cell culture. CM possess and provide key TME components that activate cancer-related pathways and drive epigenetic changes for malignant transformation of the iPSCs [13,100]. However, it is important to note that the CM-based CSCs might be able to mimic the transcriptomic landscape of native CSCs in vivo only, limiting their usefulness in in vitro studies [98,131]. While genetic manipulation of iPSCs can sporadically generate CSCs, the significance of epigenetic changes associated with CSC behavior is typically overlooked in these methods [116,151]. Contrarily, the CM-dependent conversion of iPSCs into CSCs postulates that malignant transformation is achievable without known genetic abnormalities, including key driver mutations [98,103]. However, oncogenic mutations are involved in the development of CSC features in cancer cells as revealed through the better reprogramming efficiency of p53-null or mutant liver cancer cells compared with their wild-type counterparts [145]. SW48 colorectal cancer cells with heterozygous p53 R273H missense mutation gave rise to an increased number of CSCs, signifying the contribution of an aberrant genetic makeup toward CSC development [152]. Hence, the reconciliation of these mutually exclusive CSC-induction schemes warrants an extensive characterization of genetic and microenvironment factors that govern CSC generation from normal stem cells.

A significant limitation of the in vitro functional assessment of iPSC-derived CSCs is the lack of a foolproof method for differentiating between generated CSCs and uninduced iPSCs. Sphere-formation assays are frequently employed to determine the self-renewal capacity of iPSC-derived CSCs, but they do not distinguish the tumorigenic population from uninduced iPSCs since the latter also exhibit a self-renewal ability [153]. Marker analysis provides an alternative identification approach to cancer stem cells, though their use is often contested due to an inconsistent association with CSC identity [154,155,156]. Hence, a combination of phenotypic and functional studies is recommended for CSC identification. The undefined components of CM and the sustainability of acquired CSC characteristics by iPSC-derived CSCs also present some gaps in knowledge of the CSC derivation process. An intrinsic limitation of these iPSC-derived cancer models is that only early stages of carcinogenesis can be studied with these platforms. The insufficiency regarding late-stage tumor studies may be overcome with additional TME components in three-dimensional tumor models or animal experiments.

Reprogramming cancer cells has been presented as a viable tool for generating CSC models for cancer pathway and biomarker studies and anticancer drug screening. It also offers a potent therapeutic approach due to the loss of tumorigenicity in some cases. The most significant challenge to cancer cell reprogramming is the poor efficiency of reprogramming, partly resulting from the multifaceted heterogeneity in tumor cell populations [157,158] and formidable epigenetic barriers [159]. The use of additional supporting factors, such as NANOG and LIN28, and small molecules have been shown to improve the OSKM-induced reprogramming of neoplastic cells [141,160]. However, not all cancer cell lines, even if they are from the same tissue type, are reprogrammable [16,147]. In the context of therapeutic application, epigenetic reorganization is not always sufficient in masking the functional cancer genome, and cancer traits may reappear upon differentiation [132]. Cells may acquire de novo mutations in the process of reprogramming, and genomic instability may arise in resultant cells and their derivatives depending on underlying mutations and differentiation state [161,162].

The intrinsic oncogenic potential of iPSCs presents a confounding factor in cancer-iPSC- and CSC-related studies [163,164,165,166] and needs to be addressed carefully with proper experimental controls. Interestingly, incomplete reprogramming of kidney cells through the transient expression of OSKM factors in vivo has been shown to develop Wilm’s tumor-like neoplasia, but iPSCs generated from the tumor were non-cancerous. This observation highlights the importance of thorough epigenetic remodeling for successful reprogramming [167]. Conversely, CSC features have been reported to arise in incompletely differentiated iPSCs originally generated from non-cancerous cells. This phenomenon presents a practical concern over the safe transplantation of iPSC-derived cells, even from a benign origin [168]. Alternative reprogramming approaches, such as fibromodulin protein-based reprogramming [169], for obtaining non-tumorigenic iPSC are in development. Methods for removal of undifferentiated iPSCs before transplantation to eliminate terato- or tumorigenic cells are also sought [170,171,172,173,174,175,176,177,178].

Recently, conditional reprogramming of patient-derived cancer cells has been proposed for establishing a next-generation biobank [179]. The approach aims to achieve a “reprogrammed stem-like” state through culturing patient-derived cancer cells with murine feeder cells, Swiss 3T3-J2, and ROCK inhibitor Y-27632 [179,180]. These conditionally reprogrammed cells (CRCs) maintain their original karyotype, exhibit invasive characteristics in organoid culture, show high tumorigenic potential, and demonstrate chemosensitivity to test drugs [179,181]. CRCs can be obtained from various solid tumors [181] with few exceptions of metastatic tumors [182], and they hold great promise for transforming disease modeling and drug discovery.

In conclusion, induced pluripotent stem cell technology provides an invaluable platform for the generation of versatile cancer stem-like cells for basic and translational cancer research. With the growing knowledge of CSC-inducing tumor microenvironment factors and functional genetic and epigenetic networks within cancer-propagating cells, CSC modeling strategies will be further refined for an improved recapitulation of cancer stem cell behavior. Altogether, they will inform ongoing therapeutic advancement for realizing effective therapies against refractory malignancies.

## Figures and Tables

**Table 1:** Molecular commonalities between iPSCs and cancer cells.

Protein	Putative Function in iPSCs	Function in Cancer Cells	Refs.
Oct4	Reprogramming, gene expression regulation, maintenance of pluripotency, activation of Nanog	Tumor promotion and maintenance, chemoresistance	[30,31,32,33,34,35,36,37,38,39,40]
Sox2	Cooperation with OCT4, reprogramming, gene regulation, regulation of pluripotency, activation of Nanog	Oncogenic initiation and maintenance, chemoresistance	[41,42,43,44,45]
Klf4	Stem cell renewal and maintenance, cooperation with OCT4 & SOX2, activation of Nanog	Suppression of oncogenic activity, promotion of cancer-related inflammation	[46,47,48,49,50,51,52]
c-Myc	Enhancing efficiency of iPSC generation	Tumor initiation, disruption of transcription	[53,54,55,56,57]
Nanog	Maintenance of pluripotency	Chemoresistance, cancer stem cell regeneration, hypoxia-induced angiogenesis	[58,59,60,61]
Glis1	Pro-reprogramming function	Cell growth, enhancement of invasiveness and migration	[62,63,64,65]
LIN28	Enhancement of reprogramming frequency	Oncogenic initiation and maintenance, metastasis, chemoresistance, suppression of tumor suppressor genes	[66,67,68,69,70,71]

**Table 2:** In vitro conversion of iPSCs into CSC-like cells through exposure to the tumor microenvironment.

Identity of iPSC	Cancer Cell Lines (Tissue Origin)	CSC Features Examined (Method)	Findings	Refs.
Mouse iPSCs (miPSCs)	Huh7 (liver)	CSC marker expression (RT-qPCR)	High expressions of CD24, CD133, and CD44	[97]
		In vivo tumorigenicity (inhalation and liver orthotopic injection)	miPS Huh7-CM cells gave rise to nine malignant tumors out of nine mice	
		Self-renewal potential (sphere-formation assay)	Self-renewal potential was confirmed	
		In vitro invasion and migration capacity (transwell and wound-healing assays)	Invasive ability of miPS Huh7-CM cells was enhanced upon engraftment	
miPSCs	PK-8 and KLM-1 (pancreas)	CSC marker expression (RT-qPCR)	Upregulation of CD133, CD24a, and EpCAM	[98]
		In vivo tumorigenicity (subcutaneous transplantation)	CM-based CSCs generated tumors in nine out of nine mice	
miPSCs	LLC (lung), P19 (embryonal), B16 (melanoma), MC.E12 (mammary gland)	In vivo tumorigenicity (subcutaneous transplantation)	Mouse allografts formed undifferentiated carcinomas	[13]
		Self-renewal potential (sphere-formation assay)	Self-renewal potential was confirmed	
miPSCs	LLC (lung)	In vivo tumorigenicity (subcutaneous transplantation)	Tumors derived from miPS LLC-CM grew without necrotic features	[99]
		Self-renewal potential (sphere-formation assay)	Self-renewal potential was confirmed	
miPSCs	T47D (breast), BT549 (breast)	CSC marker expression (RT-qPCR)	High expression of CD133	[100]
		In vivo tumorigenicity (subcutaneous injection)	Tumors with a high nuclear to cytoplasmic ratio and poorly-differentiated glandular structures	
		Self-renewal potential (sphere-formation assay)	Self-renewal potential was confirmed	
		In vitro invasion and migration capacity (transwell assay)	CSCcmT47D CAFLCs and CSCcm BT549 CAFLCs were highly invasive	
miPSCs	LLC (lung)	In vivo tumorigenicity (subcutaneous injection)	CSCs were capable of developing a liposarcoma that exhibited phenotypic heterogeneity	[106]
		In vitro invasion capacity (Matrigel invasion assay)	Compared with the parental miPS LLCev cells, the invasive capacities of miPS LLCevPT (primary tumor) and miPS LLCevDT (disseminated liposarcoma) cells were significantly higher	
		Self-renewal potential (sphere-formation assay)	Self-renewal potential was confirmed	
Human iPSCs (hiPSCs)	U87MG (brain)	CSC marker expression (immunocytochemistry and RT-qPCR)	Overexpression of CD133, CD44, ABCG2, and ABCC2	[109]

**Table 3:** Cancer cell reprogramming strategies and the evaluation of pluripotency induction.

Species	Cancer Cells	Reprogramming Method	Reprogramming Factors	Pluripotency Features	Tumorigenicity	Refs.
Human	HCT-15 and SK-CO-1 cell lines (colorectal adenocarcinoma)	Retroviral transduction	OCT4, SOX2, KLF4, and c-MYC	Trilineage differentiation, downregulation of pluripotency genes (incomplete reprogramming)	-	[126]
Human	Patient-derived juvenile myelomonocytic leukemia (JMML) cells	Doxycycline-inducible lentivirus	OCT4, SOX2, KLF4, and c-MYC	Endogenous pluripotency markers (NANOG, OCT4, DNMT3B, REX1), formation of three germ-cell layers in teratomas	-	[20]
Human	Patient-derived imatinib-sensitive chronic myelogenous leukemia (CML) cells	Retroviral transduction	Oct4, Sox2, Klf4, and c-Myc	Pluripotency markers (SSEA-4 and Tra-1-60), teratoma formation capacity was confirmed	-	[129]
Human	KBM7 cell line (blast crisis stage of CML)	Retroviral transduction	OCT4, SOX2, KLF4, and c-MYC	Pluripotency markers (Tra-1-81 and OCT4 and CD9), formation of all three germ-cell layers	-	[130]
Human	Patient-derived acute myeloid leukemia (AML) cells	Non-integrating Sendai virus	Oct4, Sox2, Klf4, and c-Myc	Pluripotency markers (SSEA-4 & TRA-1-81), formation of teratoma	-	[132]
Mouse	R545 cell line (melanoma)	Doxycycline-inducible lentivirus	Oct4, Klf4, and c-Myc	ESC-like colonies, demethylation of Oct4 and Nanog promoters, and teratoma formation	Chimeric mice developed from pluripotent cancer cells remained tumor-free	[24]
Human	T731 and T653 cell lines (glioblastoma)	Retroviral vector	Oct4, Sox2, and Klf4	Pluripotency marker expression	-	[141]
Human	HT-144 and A375 (melanoma cell lines), WM266.4 (*BRAF^V600D^* mutant cell line), SK-MEL147 (*NRAS* mutant cell line), Mewo (*BRAF* and *NRAS* wild-type cell line)	Lentiviral polycistronic vector	OCT4, SOX2, and KLF4	Pluripotency markers (NANOG, SOX2, and SALL4), formation of teratoma	iPCCs showed variable tumorigenicity and differentiated into non-tumorigenic lineages	[144]
Mouse	RAS+/ink4a/Arf-/- melanoma cell	Melanoma neuclei transferred to enucleated oocyte	-	-	High tumor incidence in chimeric mice	[29]
Human	DLD-1, HT-29, TE-10, MKN45, MIAPaCa-2, PANC-1, PLC, and HuCCT-1 cell lines (various gastrointestinal cancers)	Lentiviral and retroviral vectors	OCT4, SOX2, KLF4, and c-MYC	NANOG expression, Ssea-4, Tra-1-60, Tra-1-81, and Tra-2-49 surface antigens	Differentiated iPCCs showed decreased in vivo tumorigenicity	[23]
Human	SW480 and DLD-1 cell lines (colorectal cancer)	Retroviral vector	OCT4, SOX2, and KLF4	Self-renewal (in terms of CSC properties)	Sustained tumorigenicity	[21]
Human	HepG2, Hep3B, Huh7, and PLC cell lines (liver cancer)	Retroviral vector	OCT4, SOX2, KLF4, and c-MYC	Hep3B cells acquired similar characteristics to pluripotent stem cells	-	[145]
Human	PLC/PRF/5 cell line (hepatoma)	Lentiviral and retroviral vectors	OCT4, SOX2, KLF4, and c-MYC	Rex1 and Nanog expression, trilineage differentiation potential	Colony-forming tumorigenic iPCCs	[146]
Human	PANC1 cell line (pancreatic ductal adenocarcinoma)	Retroviral or lentiviral vectors	OCT4, SOX2, KLF4, and c-MYC	Endogenous Nanog and Tra-1-60 positive for ALP activity, differentiation into germ layer derivatives	-	[147]
Human	HTB-9 and T24 cell lines (bladder cancer)	Sendai virus	OCT4, SOX2, KLF4, and c-MYC	Reprogrammed T24 cells showed epithelial-like morphology, colony-forming ability, expression of pluripotency-associated markers, and differentiation capacity	-	[16]
Human	Patient-derived pancreatic ductal adenocarcinoma (PDAC) cells	Lentiviral vectors	Oct4, Sox2, Klf4, and c-Myc	Expression of pluripotency markers NANOG, OCT4, and SSEA4, teratoma formation, and expression of three germ layer markers	One iPSC line progressed from early to late stages of PDAC	[149]
Human	A549 cell line (lung carcinoma)	Lentiviral vector	Oct4 and Nanog	Stemness-related gene expression, self-renewal	In vivo tumorigenic and metastatic abilities	[150]
Human	Patient-derived plexiform neurofibroma cells	Retroviral vector	OCT4, SOX2, KLF4, and c-MYC	Pluripotency markers (TRA-1-81, SSEA3, and SSEA4), teratoma formation	*NF1*^−/−^ iPSC-derived Schwann cells exhibited a continuous high proliferation rate and formed 3D spheres	[142]

## Data Availability

This is a review paper and no new datasets were generated. The datasets used and analyzed are published in this paper and cited from the references.
